# Distinct Evolutionary Patterns of NBS-Encoding Genes in Three Soapberry Family (Sapindaceae) Species

**DOI:** 10.3389/fgene.2020.00737

**Published:** 2020-07-10

**Authors:** Guang-Can Zhou, Wen Li, Yan-Mei Zhang, Yang Liu, Ming Zhang, Guo-Qing Meng, Min Li, Yi-Lei Wang

**Affiliations:** ^1^College of Agricultural and Biological Engineering (College of Tree Peony), Heze University, Heze, China; ^2^Institute of Botany, Jiangsu Province and Chinese Academy of Sciences, Nanjing, China; ^3^State Key Laboratory of Pharmaceutical Biotechnology, School of Life Sciences, Nanjing University, Nanjing, China

**Keywords:** Sapindaceae, NBS-encoding gene, phylogeny, gene duplication/loss, evolutionary pattern

## Abstract

Nucleotide-binding site (NBS)-type disease resistance genes (*R* genes) play key roles in plant immune responses and have co-evolved with pathogens over the course of plant lifecycles. Comparative genomic studies tracing the dynamic evolution of NBS-encoding genes have been conducted using many important plant lineages. However, studies on Sapindaceae species have not been performed. In this study, a discrepant number of NBS-encoding genes were identified in the genomes of *Xanthoceras sorbifolium* (180), *Dinnocarpus longan* (568), and *Acer yangbiense* (252). These genes were unevenly distributed and usually clustered as tandem arrays on chromosomes, with few existed as singletons. The phylogenetic analysis revealed that NBS-encoding genes formed three monophyletic clades, RPW8-NBS-LRR (RNL), TIR-NBS-LRR (TNL), and CC-NBS-LRR (CNL), which were distinguished by amino acid motifs. The NBS-encoding genes of the *X. sorbifolium*, *D. longan*, and *A. yangbiense* genomes were derived from 181 ancestral genes (three RNL, 23 TNL, and 155 CNL), which exhibited dynamic and distinct evolutionary patterns due to independent gene duplication/loss events. Specifically, *X. sorbifolium* exhibited a “first expansion and then contraction” evolutionary pattern, while *A. yangbiense* and *D. longan* exhibited a “first expansion followed by contraction and further expansion” evolutionary pattern. However, further expansion in *D. longan* was stronger than in *A. yangbiense* after divergence, suggesting that *D. longan* gained more genes in response to various pathogens. Additionally, the ancient and recent expansion of CNL genes generated the dominance of this subclass in terms of gene numbers, while the low copy number status of RNL genes was attributed to their conserved functions.

## Introduction

Nucleotide-binding site (NBS)-encoding genes are the largest type (∼80%) of disease resistance genes (*R* genes) found in plants and are responsible for the protection against various pathogens (Liu et al., 2007; Dangl et al., 2013). A typical NBS-encoding gene consists of a variable domain at the N-terminal, a highly conserved NBS domain in the middle, and a diverse leucine-rich repeat (LRR) domain at the C-terminal. Therefore, according to the structure of N-terminal domains that possess a coiled-coil (CC), *Drosophila* toll and mammalian interleukin-1 receptor-like (TIR), or resistance to powdery mildew8 (RPW8) domain, NBS-encoding genes can be classified into three subclasses: CC-NBS-LRR (CNL), TIR-NBS-LRR (TNL), and RPW8-NBS-LRR (RNL) (Meyers et al., 2003; Shao et al., 2014, 2016, 2019; Zhang et al., 2016, 2020; Qian et al., 2017; Xue et al., 2020). As an example of function, the CNL gene, *RPP8*, in *Arabidopsis thaliana* provides resistance against downy mildew after *Peronospora parasiticia* infection (Mcdowell et al., 1998). Another CNL gene, *Pik*, confers resistance to rice blast caused by *Magnaporthe grisea* infection (Zhai et al., 2011). The tobacco TNL gene, *N*, prevents tobacco mosaic virus invasion (Whitham et al., 1994). Functionally, CNL and TNL genes act as detectors that recognize specific pathogen effectors encoded by avirulence genes and initiate downstream hypersensitive reactions in the resistance pathway (Dangl and Jones, 2001; Mchale et al., 2006), while RNL genes appear to function downstream and transduce signals from CNL or TNL genes through interactions with corresponding partners (Bonardi et al., 2011; Collier et al., 2011; Tamborski and Krasileva, 2020). Therefore, NBS-encoding *R* genes are of great importance to plant growth and would tangibly benefit humankind if properly used in disease resistance breeding.

NBS-encoding genes constitute a large gene family found in plant genomes. With the accumulation of more plant whole-genome sequences, genome-wide evolutionary analyses of NBS-encoding genes have been performed in many plants since the first comprehensive study on NBS-encoding genes in *A. thaliana* (Meyers et al., 2003). Furthermore, comparative genomic studies on the NBS-encoding gene family have been performed among a few closely related plant species, which exhibited different evolutionary patterns. For example, frequent gene losses and limited gene duplications resulted in small number of NBS-encoding genes in three Cucurbitaceae species (Lin et al., 2013). Both Li et al. (2010) and Luo et al. (2012) investigated the NBS-encoding genes in four Poaceae species, including rice, maize, sorghum, and brachypodium, which exhibited a “contraction” evolutionary pattern and may have been caused by gene losses or frequent gene deletions and translocations. Similar analyses were performed on Fabaceae, Rosaceae, and Brassicaceae species, of which Fabaceae and Rosaceae species exhibited a “consistent expansion” evolutionary pattern (Shao et al., 2014; Jia et al., 2015), while five Brassicaceae species exhibited a “first expansion and then contraction” evolutionary pattern (Zhang et al., 2016). Moreover, although plants belonged to the same family, the evolutionary patterns of NBS-encoding genes were also diverse. For example, in three Solanaceae crop species, pepper exhibited a “contraction” pattern, tomato showed a “first expansion and then contraction” pattern, and potato presented a “consistent expansion” pattern (Qian et al., 2017). In four orchid species, *Phalaenopsis equestris* and *Dendrobium catenatum* exhibited an “early contraction to recent expansion” evolutionary pattern, while *Gastrodia elata* and *Apostasia shenzhenica* showed a “contraction” evolutionary pattern (Xue et al., 2020). Recently, a large scale analysis of the NBS-encoding genes in 22 representative angiosperms demonstrated that CNL genes exhibited a “gradual expansion” evolutionary pattern during the first 100 million years of angiosperm evolution, then underwent intense expansion along with TNL genes, which corresponded with the explosion of fungal diversity (Shao et al., 2016).

The soapberry family (Sapindaceae) consists of 135 genera and ∼1500 species, which is comprised mostly of trees or shrubs and some herbaceous climbers, which are widely distributed throughout the tropics and subtropics, especially in Southeastern Asia (Flora of China^1^). Sapindaceae species possess many great economic uses. For example, the seed kernels of Yellowhorn (*Xanthoceras sorbifolium*), a major woody oil plant species, contains as much as 67% oil (Venegas-Calerón et al., 2017), and extractions from its husks improve learning and memory that could be used to treat Alzheimer’s disease (Ji et al., 2017; Zhang et al., 2018). Longan (*Dinnocarpus longan*), an important evergreen fruit tree, is mainly grown in Southern China and serves as a source of traditional medicine and timber (Chung et al., 2010; Mei et al., 2014). *Acer yangbiense* is a charismatic landscape plant that possesses a colorful foliage is a newly described critically endangered endemic maple tree found in Southwestern China (Chen et al., 2003), and it possesses many bioactive compounds (Bi et al., 2016; Yang et al., 2019).

Recently, the high-quality genome sequences of *X. sorbifolium*, *D. longan*, and *A. yangbiense* were made available and all recovered >94% complete BUSCO genes by BUSCO analysis (Lin et al., 2017; Bi et al., 2019; Yang et al., 2019). An analysis of the NBS-encoding genes in *D. longan* was performed, and researchers found high number and recent expansions/contractions of these genes that may be attributed to the genomic basis for insect, fungus, and bacteria resistance (Lin et al., 2017). However, these findings require further elucidation of this phenomenon caused by gene duplication/loss events. Additionally, the systematic evaluation and comparison of NBS-encoding genes at the genome level in more Sapindaceae species is needed to obtain a better understanding of this family’s molecular evolutionary history. In this study, *X. sorbifolium*, *D. longan*, and *A. yangbiense* genome sequence data were utilized to perform comparative genomic analyses in order to uncover the evolutionary features and patterns of NBS-encoding genes in the Sapindaceae family, as well as further investigate the mechanisms of these evolutionary changes.

## Materials and Methods

### Identification and Classification of the NBS-Encoding Genes

The whole genomes of the *X. sorbifolium*, *D. longan*, and *A. yangbiense* were used in this study (Supplementary Figure S1). Genomic sequences and annotation files were obtained from the GigaScience database (*X. sorbifolium*^2^, Bi et al., 2019; *D. longan*^3^, Lin et al., 2017; *A. yangbiense*^4^, Yang et al., 2019). The method for NBS-encoding gene identification was described in a previous study (Shao et al., 2016). Briefly, BLAST and hidden Markov model (HMM) searches were simultaneously conducted using the NB-ARC domain (Pfam accession No.: PF00931) as the query sequence to identify candidate NBS-encoding genes in the three genomes. The threshold expectation value was set to 1.0 for the BLAST search. For the HMM search^5^, default settings were used. Then, the identified candidate sequences were merged and the redundant hits were removed. In order to confirm the presence of the NBS domain, the remaining sequence hits were subjected to online Pfam analysis^6^ with an E-value of 10^–4^. All of the identified NBS-encoding genes were subjected to NCBI’s conserved domain database^7^ using the default settings to determine whether they encoded CC, TIR, RPW8, or LRR domains.

### Chromosomal Distribution and Cluster Arrangement of Identified NBS-Encoding Genes

The chromosomal locations of all identified NBS-encoding genes in Sapindaceae genomes were determined by retrieving relevant information from the downloaded gff files. Gene cluster was determined according to the criterion used for *Medicago truncatula* (Ameline-Torregrosa et al., 2008): if two neighboring NBS-encoding genes were located within 250 kb on a chromosome, these two genes were regarded as members of the same gene cluster. Based on this criterion, the NBS-encoding genes in Sapindaceae genomes were assigned to clustered loci and singleton loci, which were mapped along the chromosomes.

### Sequence Alignment and Conserved Motif Analysis of the NBS Domain

Amino acid sequences of the NBS domain were extracted from the identified NBS-encoding genes and used for multiple alignments using ClustalW integrated in MEGA 7.0 using the default settings (Kumar et al., 2016). Sequences that were too short (<190 amino acids, less than two-thirds of a regular NBS domain) or too divergent were removed to prevent interference with the alignments and subsequent phylogenetic analysis. The resulting alignments were manually corrected and improved using MEGA 7.0. The conserved protein motifs within the NBS domain of the three subclasses of NBS-encoding genes were analyzed by Multiple Expectation Maximization for Motif Elicitation (MEME) and WebLogo using the default settings (Crooks et al., 2004; Bailey et al., 2006). Additionally, structural motif annotation was performed using the Pfam analysis^8^ and SMART tools^9^.

### Phylogenetic and Gene Loss/Duplication Analysis of the NBS-Encoding Genes

To explore the relationships of NBS-encoding genes in the three Sapindaceae genomes, a phylogenetic tree was constructed based on the amino acid sequences of the conserved NBS domain using the NBS-encoding genes of *A. thaliana* as a reference. The NBS-encoding genes of *A. thaliana* were identified using the same method, and these NBS-encoding genes were also used in a previous study (Zhang et al., 2011). Amino acid sequences were aligned as described above. The phylogenetic tree was constructed using IQ-TREE and the maximum likelihood method based on the best-fit model estimated by ModelFinder (Nguyen et al., 2015; Kalyaanamoorthy et al., 2017); branch support values were assessed using UFBoot2 tests (Minh et al., 2013). The short or divergent sequences that were removed from the phylogenetic analyses were BLASTp searched against all of the identified NBS genes to identify their potential phylogenetic positions by identifying their closest relatives. Additionally, in order to identify the gene duplication/loss events during the speciation of the three Sapindaceae species, the NBS-encoding gene phylogenetic tree was reconciled with the real species tree using Notung software (Stolzer et al., 2012). The NBS-encoding genes on the tree that formed a monophyletic branch and originated from one ancestral gene inherited from the common ancestor of the three Sapindaceae species was defined as a Sapindaceae lineage gene. One or more Sapindaceae lineage genes that formed a monophyletic branch with the *A. thaliana* NBS-encoding genes were inherited from a common ancestor of the three Sapindaceae species and *A. thaliana*, and were defined as Malvids lineage genes.

### Synteny Analyses Across/Within Sapindaceae Genomes and Gene Duplication Type Determination

The MCScanX package (Lee et al., 2012; Wang et al., 2012) was used to identify syntenic blocks within a genome or between different genomes through pair-wise all-against-all blast of protein sequences. The purposes of synteny analysis were to explore the pattern of conservation of NBS-encoding gene loci among the Sapindaceae genomes and determine the types of NBS-encoding gene duplication. Synteny relationship of NBS-encoding genes was displayed by TBtools^10^.

## Results

### Identification and Classification of the NBS-Encoding Genes

A total of 180, 252, and 568 non-redundant NBS-encoding genes were identified from the genomes of *X. sorbifolium*, *A. yangbiense*, and *D. longan*, respectively (Table 1 and Supplementary Table S1), accounting for 0.73, 0.89, and 1.83% of the 24,672, 28,320, and 31,007 annotated protein-coding genes in *X. sorbifolium*, *A. yangbiense*, and *D. longan* genomes, respectively (Lin et al., 2017; Bi et al., 2019; Yang et al., 2019). The average lengths of NBS-encoding genes in *X. sorbifolium*, *D. longan*, and *A. yangbiense* were 6081, 5569, and 6199 bp, while the average lengths of the coding region of NBS-encoding genes in *X. sorbifolium*, *D. longan*, and *A. yangbiense* were 3082, 3132, and 3142 bp, respectively. *D. longan* possessed the largest number of NBS-encoding genes and was 3.16- and 2.25-times greater than *X. sorbifolium* and *A. yangbiense*, respectively. The identified NBS-encoding genes from the three Sapindaceae species were divided into the CNL, TNL, and RNL subclasses based on their domain compositions and primary phylogenies. Among the three subclassses, CNL genes overwhelmingly outnumbered TNL and RNL genes with proportions of 81.11, 89.26, and 89.68% in *X. sorbifolium*, *D. longan*, and *A. yangbiense*, respectively. The proportion of TNL genes was 17.78, 9.15, and 7.94% in *X. sorbifolium*, *D. longan*, and *A. yangbiense*, respectively, while the number of RNL genes was the smallest with only two, nine, and six genes, respectively. Not all of the identified NBS-encoding genes had intact structures possessing all three domains (CC/TIR/RNL) with many either lacking the CC/TIR/RPW8 domain at the N-terminus, the LRR domain at the C-terminus, or domains at both termini (Table 1). Additionally, some identified NBS-encoding genes were classified as “other” in TNL and CNL due to their atypical structural domain compositions (Table 1). For example, the *D. longan* genome encoded two “other” genes in TNL, including LN_TIR_L and TLTN, and 13 “other” genes in CNL, including CNLNL(2), N_CC_LN_CC_LN_CC_L(1), N_CC_N_CC_LN_CC_L(1), NLCNL(1), CNLCNL(4), CNLN(1), N_CC_N_CC_L(2), and CNN(1).

**TABLE 1:** The number of identified NBS-encoding genes in the three Sapindaceae genomes.

Domain compositions	*X. sorbifolium*	*A. yangbiense*	*D. longan*
**TNL subclass**	**32 (17.78%)**	**20 (7.94%)**	**52 (9.15%)**
*TNL* (intact)	24	3	24
*TN*	0	0	7
*NL*	5	10	6
*N*	3	4	13
*Other*	0	3	2
**CNL subclass**	**146 (81.11%)**	**226 (89.68%)**	**507 (89.26%)**
*CNL* (intact)	55	26	218
*CN*	27	30	88
*NL*	45	95	107
*N*	18	72	81
*Other*	1	3	13
**RNL subclass**	**2 (1.11%)**	**6 (2.38%)**	**9 (1.58%)**
*RNL* (intact)	2	2	5
*RN*	0	1	0
*NL*	0	1	1
*N*	0	2	3
*Other*	0	0	0
**Total number**	**180**	**252**	**568**
Proportion to total protein-coding genes	0.73%	0.89%	1.83%
Average gene size (bp)	6081	6199	5569
Average length of coding sequence (bp)	3082	3142	3132

### Distribution and Organization of NBS-Encoding Genes in Sapindaceae Genomes

The NBS-encoding genes were unevenly distributed among different chromosomes. For example, Chrom (chromosome) 9 of *X. sorbifolium* genome contains the most genes (42 genes), whereas the Chrom 1 and 11 contain the fewest (only two genes in each Chrom) (Supplementary Figure S3). In *A. yangbiense*, Chrom 3 contains the most genes (89 genes), whereas no NBS-encoding gene was detected on Chrom 11 (Supplementary Figure S4). Uneven distributions were also observed among the three subclasses of NBS-encoding genes (Supplementary Figures S3, S4). Chrom 9 (41 genes) of *X. sorbifolium* and Chrom 3 (86 genes) of *A. yangbiense* contained the most CNL genes, whereas Chrom 4 (20 genes) of *X. sorbifolium* and Chrom 2 (five genes) and 8 (five genes) of *A. yangbiense* contained the most TNL genes. All chromosomes of the two species contained CNL genes except Chrom 10 and 11 of *A. yangbiense*, whereas only seven chromosomes of *X. sorbifolium* and six chromosomes of *A. yangbiense* contain TNL genes. There were too few RNL genes for this analysis. The majority of NBS-encoding genes were organized into clusters rather than singletons in *X. sorbifolium* and *A. yangbiense* genomes, and their ratios were 2.98 and 5.31, respectively (Table 2). *A. yangbiense* contained more clustered genes than *X. sorbifolium* (207 genes vs 131 genes), and the largest gene cluster of *X. sorbifolium* and *A. yangbiense* were on Chrom 14 (14 genes) and Chrom 3 (21 genes), respectively. Since the NBS-encoding genes in *D. longan* genome were not anchored to chromosomes yet, the chromosomal locations and cluster assignments of these genes were not examined.

**TABLE 2:** Organization of NBS-encoding genes in the three Sapindaceae genomes.

Loci and genes	*X. sorbifolium*	*A. yangbiense*	*D. longan*
No. of chromosome-anchored loci (and genes)	83 (175)	77 (246)	/
No. of singleton loci (no. of genes)	44 (44)	39 (39)	/
No. of clustered loci (no. of genes)	39 (131)	38 (207)	/
Clustered genes/singleton genes	2.98	5.31	/
Average no. of genes in clusters	3.36	5.45	/
No. of clusters with 10 or more genes	1	7	/
No. of genes in the largest cluster	14 (Chrom 14)	21 (Chrom 3)	/

*No., number; Chrom., chromosome; /, not detected.*

### Motif Analysis of the NBS Domain

**FIGURE 1:**
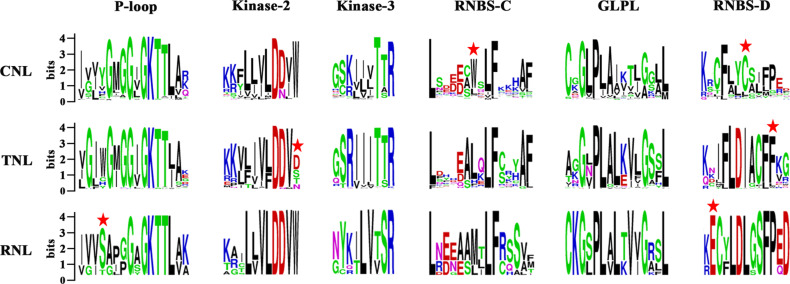
Six conserved motifs of NBS domains in the three Sapindaceae species. Amino acids of the six conserved motifs were extracted. Different conserved amino acids among CNL, TNL, and RNL subclass genes are labeled with a red star. Details of the amino acids of the whole NBS domain are presented in Supplementary Figure S2.

The NBS domain consists of several functional motifs that were conserved and strictly ordered among the NBS-encoding genes (Yue et al., 2012). A total of six conserved motifs were identified in each subclass of the NBS domains in the three Sapindaceae species using MEME and WebLogo (Crooks et al., 2004; Bailey et al., 2006). From the N-terminus to the C-terminus, these motifs included the P-loop, Kinase-2, Kinase-3, RNBS-C, GLPL, and RNBS-D motifs. Comparisons of the amino acid sequences of these motifs are presented in Figure 1. The P-loop, Kinase-2, Kinase-3, and GLPL motifs exhibited high similarity among the three subclasses of NBS-encoding genes, suggesting that the NBS domains with critical functions regulating the immune responses were homologous. The other two motifs, especially RNBS-D, were poorly conserved among the three subclasses of NBS-encoding genes. The variation of these motifs may be responsible for further functional divergences within these subclasses. The subclass-specific signatures within the six motifs were further analyzed, which could be used as preliminary labels to identify CNL, TNL, or RNL genes. For example, Tryptophan (W) at the seventh position of the RNBS-C and Cysteine (C) at the seventh position of RNBS-D in CNL genes, phenylalanine (F) at the 11th position of the RNBS-D in TNL genes, and serine (S) at the fourth position of the P-loop and glutamic acid (E) at the second position of the RNBS-D in RNL genes. Additionally, the amino acids at the final position of Kinase-2 could also be used to distinguish TNL genes from CNL and RNL genes (Figure 1). Therefore, the subclass of a given NBS-encoding gene could be classified based on the amino acid signatures of the motif sequences.

### Phylogenetic Analysis of the NBS-Encoding Genes

**FIGURE 2:**
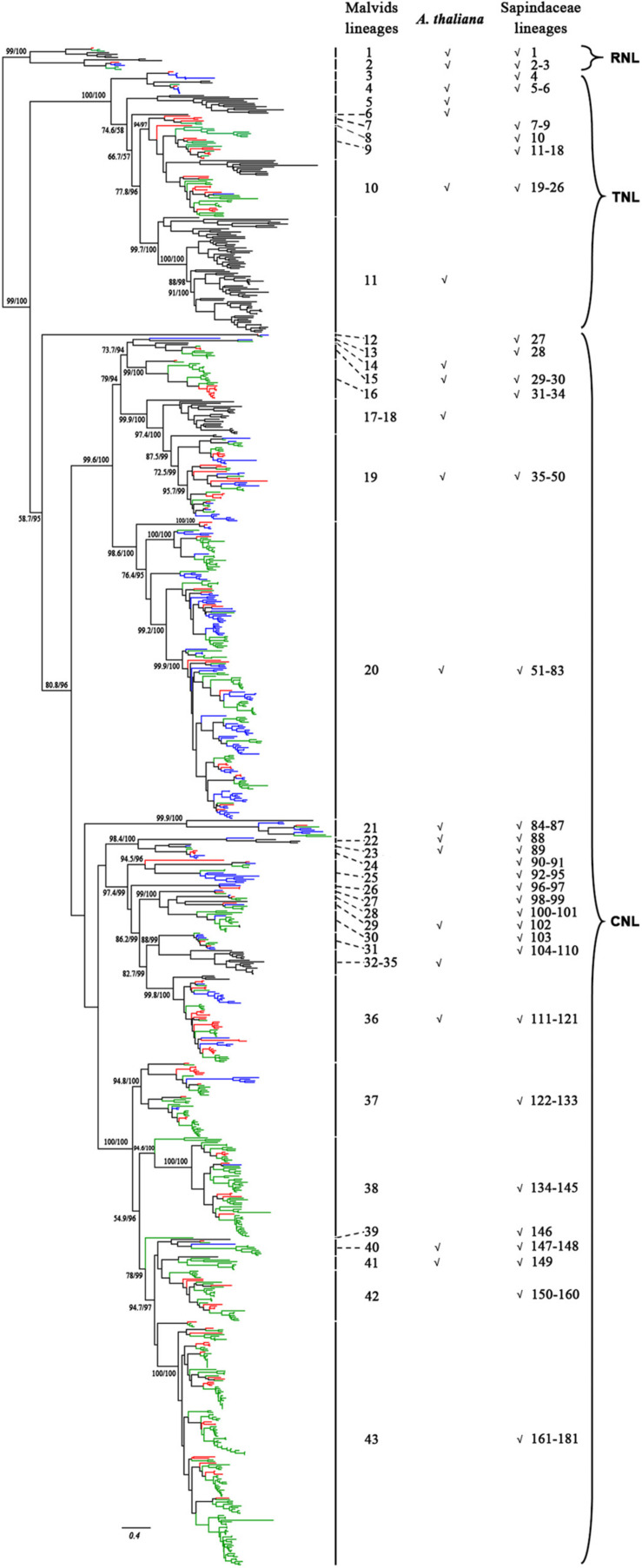
Phylogenetic relationships of the NBS-encoding genes in *X. sorbifolium*, *A. yangbiense*, and *D. longan* based on the amino acids of conserved NBS domains. Red, blue, green, and black lines represent the NBS-encoding genes in *X. sorbifolium*, *A. yangbiense*, *D. longan*, and *A. thaliana*, respectively. Support values > 70% for basal nodes are shown. The reconstructed phylogeny was divided into 43 Malvids and 181 Sapindaceae lineage NBS-encoding genes. The first column number 1–43 represents the Malvids lineage NBS-encoding genes, and the last column number 1–181 represents the Sapindaceae lineage NBS-encoding genes. The presence of Malvids lineage NBS-encoding genes in *A. thaliana* and the common ancestor of the three Sapindaceae species is indicated by “√.” The scale bar indicated amino acid substitutions/sites. The detailed phylogenetic tree of the NBS-encoding genes, including gene names, evolutionary relationships among genes, and supporting values of all of the nodes, is presented in Supplementary Figure S5.

To reconstruct the phylogenetic relationship of the NBS-encoding genes in Sapindaceae, a phylogenetic tree was constructed based on the amino acid sequences of NBS domain alignments using the NBS-encoding genes of *A. thaliana* as a reference. To attain better phylogeny, too short or extremely divergent NBS domains were removed from the data matrix. A total of 957 genes (*X. sorbifolium*, 159; *D. longan*, 468; *A. yangbiense*, 173; *A. thaliana*, 157) were obtained and used to reconstruct the evolutionary history of the NBS-encoding genes. The phylogenetic tree was composed of three monophyletic clades, RNL, TNL, and CNL, with support values > 99%; many internal nodes had high (>70%) support values (Figure 2 and Supplementary Figure S5). The three clades represented the divergence of RNL, TNL, and CNL genes. Compared to TNL and CNL, the branch lengths of RNL genes were short (Figure 2), suggesting that they had a low evolutionary rate.

To gain insight on the evolution of the NBS-encoding genes before and after divergence of *A. thaliana* and the three Sapindaceae species, Notung software was used to reconcile gene duplication/loss events of the NBS-encoding genes at each node of the phylogenetic tree (Stolzer et al., 2012). Based on the definitions (refer to section “Materials and Methods” for details), 43 Malvids lineage genes were retrospected (Figure 2). The ratio of RNL:TNL:CNL genes was 2:9:32. Among them, only 13 genes (two RNL, two TNL, and nine CNL) were reserved by both *A. thaliana* and the common ancestor of the three Sapindaceae species, while 19 genes (four TNL, 15 CNL) were lost by *A. thaliana* and 10 genes (three TNL, seven CNL) were lost by the common ancestor.

**FIGURE 3:**
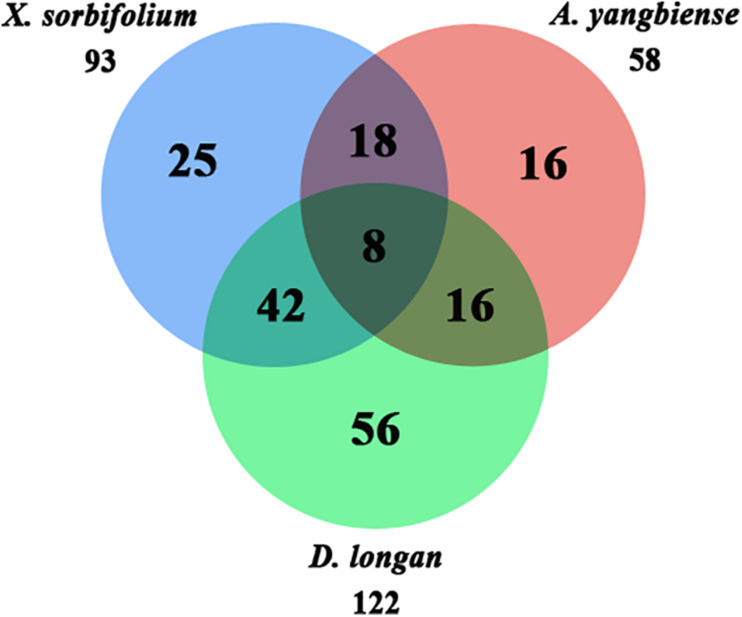
Ancestral Sapindaceae linage genes in the *X. sorbifolium*, *A. yangbiense*, and *D. longan* genomes.

A total of 33 Malvids lineage genes (two RNL, six TNL, 25 CNL) were inherited by the common ancestor of the three Sapindaceae species and these genes intensively expanded to 181 Sapindaceae lineage genes (three RNL, 23 TNL, 155 CNL) (Figure 2). CNL genes exhibited a more active expansion rate than RNL or TNL genes, especially the CNL Malvids lineage 19, 20, and 43 expanded 16, 33, and 21 Sapindaceae lineages, respectively (Figure 2). Further analysis revealed that 93, 122, and 58 of 181 Sapindaceae lineages were inherited by *X. sorbifolium*, *D. longan*, and *A. yangbiense*, respectively (Figure 3), while only eight lineages were reserved in all three genomes. A total of 76 Sapindaceae lineages (*X. sorbifolium* and *D. longan*, 42; *D. longan* and *A. yangbiense*, 16; *X. sorbifolium* and *A. yangbiense*, 18) were maintained in only two genomes and 97 lineages were species-specific (*X. sorbifolium*, 25; *D. longan*, 56; *A. yangbiense*, 16). The distribution patterns suggest that these ancestral gene lineages have experienced differential gene duplication/loss events when the three Sapindaceae species diverged.

### Syntenic Analysis of NBS-Encoding Genes in Sapindaceae Genomes

**FIGURE 4:**
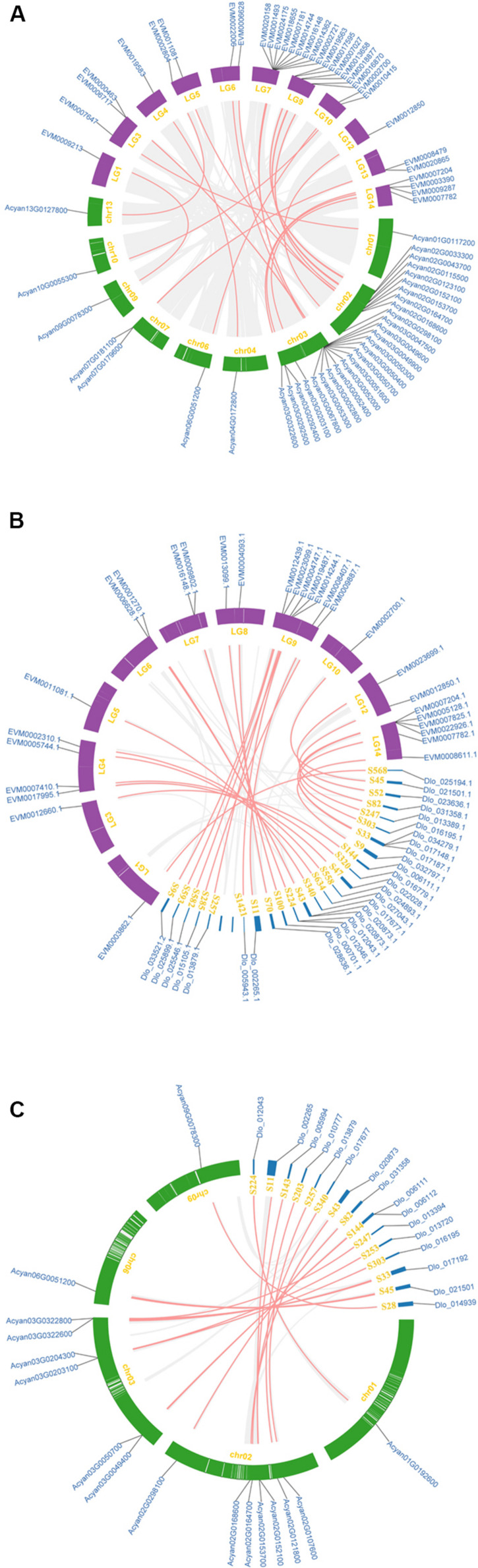
Synteny of the three Sapindaceae species NBS-encoding genes. **(A)** Synteny of *X. sorbifolium* and *A. yangbiense* NBS-encoding genes. **(B)** Synteny of *X. sorbifolium* and *D. longan* NBS-encoding genes. **(C)** Synteny of *A. yangbiense* and *D. longan* NBS-encoding genes. LG1-15, *X. sorbifolium* chromosomes; chr01-13, *A. yangbiense* chromosomes; S, *D. longan* scaffolds. Red and gray lines represent synteny NBS-encoding genes and non-NBS-encoding genes, respectively.

The synteny analysis was performed between and within the three Sapindaceae genomes (the *D. longan* genome was presented as scaffold form). A total of 33 syntenic NBS-encoding genes were detected between *X. sorbifolium* and *A. yangbiense* genome (Figure 4A and Supplementary Table S2). Among them, the number of syntenic CNL and RNL subclass genes was 32 and one, respectively, whereas no syntenic TNL subclass genes were detected. The CNL, TNL, and RNL subclass syntenic genes were all detected between *X. sorbifolium* and *D. longan* genome (Figure 4B and Supplementary Table S2), and the gene number was 26, 2, and 1, respectively. However, there were only 16 CNL subclass syntenic genes were detected between *A. yangbiense* and *D.longan* (Figure 4C and Supplementary Table S2). Moreover, there were only seven syntenic NBS-encoding genes preserved by the three genomes and they all belong to the CNL subclass (Supplementary Table S2). The syntenic NBS-encoding genes revealed three different forms: singleton to singleton, singleton to cluster, and cluster to cluster. These forms were resulted from different extents of gene duplication/loss of the ancestor genes. The large number of NBS-encoding genes showing presence/absent (P/A) polymorphism and asymmetric gene numbers among co-linear loci indicate that the NBS-encoding genes have experienced dramatic gain and loss during the evolution of Sapindaceae species.

There were three types of NBS-encoding gene duplications: local tandem duplication, dispersed (or ectopic) duplication, and whole genome duplications (WGDs) or segmental duplication (Leister, 2004). MCScanX software was adopted to determine the type of gene duplications in producing NBS-encoding genes during the evolution of Sapindaceae species. As shown in Table 3, tandem and dispersed duplication were the main contributors to gene expansion events in *X. sorbifolium* and *A. yangbiense* genomes. However, the proportion of segmental duplicated genes might have been underestimated because of the syntenic relationship of NBS-encoding genes would be disrupted during long-term evolution. The gene duplication types of the NBS-encoding genes in *D. longan* genome were not examined, since these genes were not anchored to chromosomes yet.

**TABLE 3:** Contributions of three duplication types in producing NBS-encoding genes during the evolution of Sapindaceae species.

Different types of duplication	*X. sorbifolium*	*A. yangbiense*	*D. longan*
Total no. of NBS-encoding genes	180	252	568
Local tandem duplication	108 (60%)	172 (68.2%)	/
Dispersed duplication	55 (30.6%)	61 (24.2%)	/
WGD or segmental duplication	12 (6.7%)	13 (5.2%)	/
Unanchored genes	5 (2.7%)	6 (2.4%)	568

*No., number; /, not detected.*

### Gene Duplications/Losses Resulting in Dynamic Evolutionary Patterns of the NBS-Encoding Genes

**FIGURE 5:**
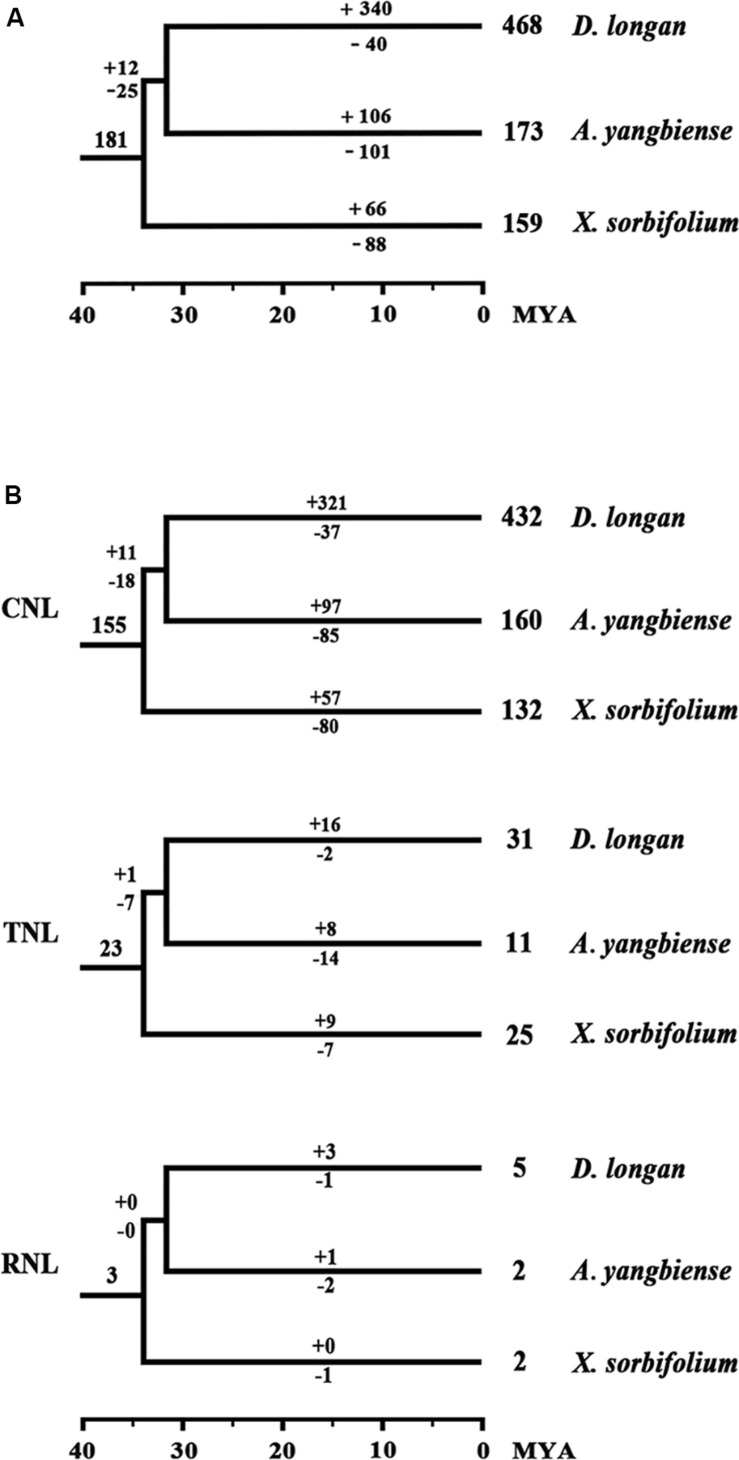
Duplication/loss events of the NBS-encoding genes during the speciation of *X. sorbifolium*, *A. yangbiense*, and *D. longan*. **(A)** Differential gene duplication and loss events of NBS-encoding genes. **(B)** Duplication/loss events of CNL, TNL, and RNL genes. Gene duplication/loss events are indicated by numbers with “+” or “−” on each branch, respectively. Detailed information for the duplication/loss events of the NBS-encoding genes are presented in Supplementary Figure S6.

To assess the evolutionary patterns of the NBS-encoding genes during the speciation of the three Sapindaceae species, the gene duplication/loss events of the NBS-encoding genes were restored in the phylogenetic tree based on the conserved NBS domain sequences. The retrospective analysis revealed that 43 ancient NBS-encoding genes were shared by the common ancestor of *A. thaliana* and the three Sapindaceae species (Figure 2). Among these ancient genes, 33 were inherited and duplicated into 181 genes in the common ancestor of the three Sapindaceae species. These 181 ancestral Sapindaceae genes experienced different evolutionary patterns, which were reflected by species-specific gene duplication/loss events during the speciation of *X. sorbifolium*, *D. longan*, and *A. yangbiense. X. sorbifolium* duplicated 66 genes (57 CNL, nine TNL) and lost 88 genes (80 CNL, seven TNL, one RNL) during speciation (Figures 5A,B and Supplementary Figure S6), resulting in a slight decrease in the number of the NBS-encoding genes in its genome. Similarly, the common ancestor of *D. longan* and *A. yangbiense* after diverging from *X. sorbifolium* lost 25 genes (18 CNL, seven TNL) and duplicated 12 genes (11 CNL, one TNL), resulting in a decrease in the total number of genes (168). However, *D. longan* and *A. yangbiense* underwent considerably different evolutionary processes after divergence (Figures 5A,B and Supplementary Figure S6). Specifically, *D. longan* duplicated 340 genes (321 CNL, 16 TNL, three RNL), but lost 40 genes (37 CNL, two TNL, one RNL), thus, the gene number clearly increased in its genome. *A. yangbiense* duplicated 106 genes (97 CNL, eight TNL, one RNL) and lost 101 genes (85 CNL, 14 TNL, two RNL), resulting in a slight increase in the number of NBS-encoding genes in its genome. Therefore, the NBS-encoding genes in the three Sapindaceae species exhibited dynamic and distinct evolutionary patterns.

## Discussion

### Copy Number Variation of the NBS-Encoding Genes Among Different Species

The NBS-encoding gene family is one of the most divergent plant genome families with high copy number discrepancies among plant lineages/species (Jacob et al., 2013). For example, several NBS-encoding gene sequences were identified in some orchid species genomes (Xue et al., 2020), which possess the smallest number of genes observed thus far. Dozens of NBS-encoding genes have been identified in papaya, melon, cucumber, and watermelon (Lin et al., 2013; Zhang et al., 2016). Hundreds of gene copies in *Arabidopsis*, rice, potato, tomato, pepper, cotton, soybean, grape, poplar, barley, and sunflower have also been identified (Yang et al., 2008; Li et al., 2010; Guo et al., 2011; Wei et al., 2013; Shao et al., 2014; Wu et al., 2014; Andersen et al., 2016; Qian et al., 2017; Neupane et al., 2018). Moreover, 1219 and 1303 copies were identified in wheat and apple genomes, respectively (Jia et al., 2013, 2015). In this study, the gene number variation among the three Sapindaceae species was also quite large, with 180, 568, and 252 genes identified in *X. sorbifolium*, *D. longan*, and *A. yangbiense*, respectively. The proportion of NBS-encoding genes to all predicted genes in the three Sapindaceae genomes (0.73–1.83%) was higher than the proportions reported for Cucurbitaceae species (0.19–0.27%), similar to Rosaceae (0.78–2.05%) and Solanaceae (0.73–1.15%) species (Jia et al., 2015; Qian et al., 2017). The discrepancy of NBS-encoding genes among the three species should not be the result of ploidy variation, because all of the three analyzed species are diploids and have similar chromosomal numbers (Lin et al., 2017; Bi et al., 2019; Yang et al., 2019). Actually, a previous study has shown that no correlation exists between the NBS-encoding genes and species phylogeny or genome size (Jacob et al., 2013). For example, in Fabaceae, soybean (465) possessed fewer NBS-encoding genes than *M. truncatula* (571), although the genomes size of the former was twice that of the latter (Shao et al., 2014). In Brassicaceae, the numbers of NBS-encoding genes were similar across some species even though these species exhibited variations in genome size (Zhang et al., 2016).

The *A. yangbiense* genome was sequenced using the combined Pacific Biosciences Single-molecule Real-time, Illumina HiSeq X, and Hi-C technologies, and BUSCO analysis recovered 95.5% complete BUSCO genes (Yang et al., 2019); the *X. sorbifolium* genome was sequenced using the combined Illumina HiSeq, Pacific Biosciences Sequel, and Hi-C technologies, and BUSCO analysis recovered 94.7% complete BUSCO genes (Bi et al., 2019); for *D. longan* genome sequencing: using the standard Illumina library preparation protocols, and BUSCO analysis recovered 94% complete BUSCO genes (Lin et al., 2017). Additionally, the assembly quality of *D. longan* genome sequence was assessed by aligning the scaffolds to a *D. longan* transcriptome assembly from the NCBI Sequence Read Archive (SRA; SRA050205). Of the 96,251 *D. longan* transcriptome sequences reported previously (Lai and Lin, 2013), 97.55% were identified in the genome assembly (Lin et al., 2017). Although all of the three analyzed genomes are of high quality (all recovered > 94% complete BUSCO genes) according to the BUSCO analysis of the genome papers (Lin et al., 2017; Bi et al., 2019; Yang et al., 2019), the *D. longan* genome assembly has not reached a chromosomal level. A previous study revealed that genome sequence and annotation quality may affect the NBS-encoding gene identification (Bayer et al., 2018). This raises a possibility that the fragmented assembled genome of *D. longan* may have contributed to its high NBS-encoding gene number by splitting of one NBS-encoding gene into multiple genes during annotation. However, this possibility was largely rejected by coding sequence length analysis, which revealed that the average coding sequence length of NBS-encoding genes in *D. longan* is larger than that of *X. sorbifolium*. Furthermore, a close look at NBS-encoding gene profiles in the three species revealed that the number of intact NBS-encoding genes in *D. longan* (247) is even larger than the total NBS-encoding gene number in *X. sorbifolium* (180) and close to the total NBS-encoding gene number in *A. yangbiense*. Therefore, the relatively larger number of NBS-encoding genes in *D. longan* is more likely a consequence of evolution rather than other artificial reasons.

The rapid evolutionary feature of the NBS-encoding gene family caused by frequent gene duplications/loss events could elicit copy number discrepancies. The phylogenetic analysis and ancestral gene classification results of the NBS-encoding genes indicated that species-specific gene duplication/loss events occurred after species diverged from the common ancestor (Figures 2, 5A and Supplementary Figure S6). Lots of *D. longan*-specific lineage genes were found in *D. longan* and these specific lineage genes experienced recent sharp expansions by more frequent gene duplications and less gene loss events (+340 vs. -40) after the divergence of *D. longan* from *A. yangbiense* (Figure 4). The differences between gene duplication/loss events in *X. sorbifolium* (+66 vs. -88) or *A. yangbiense* (+106 vs. -101) were not as large (Figure 5A). Therefore, a copy number discrepancy of NBS-encoding genes was observed among the three Sapindaceae species. The NBS-encoding gene number variations caused by independent gene duplication/loss events were common in Cucurbitaceae, Fabaceae, Rosaceae, Poaceae, Brasssicaceae, Solanaceae, and orchid species (Li et al., 2010; Lin et al., 2013; Shao et al., 2014; Jia et al., 2015; Zhang et al., 2016; Qian et al., 2017; Xue et al., 2020). The high copy number of NBS-encoding genes that resulted from recent expansions in *D. longan* could represent candidate resistance genes that responded to various pathogens, such as witches broom disease (Lai et al., 2000; Guo, 2002). From another perspective, high copy number of NBS-encoding genes might be a disadvantage for plants in the absence of corresponding pathogens due to the fitness cost of resistance genes (Tian et al., 2003). There should be some balancing mechanisms between resistance and fitness cost provided by the large number of NBS-encoding genes in *D. longan* genome.

Chromosomal distribution analysis of NBS-encoding genes suggested an uneven distribution pattern among different chromosomes (Supplementary Figure S3, S4). The uneven distributing pattern was commonly observed in other lineages, like legume, Brassicaceae, Solanaceae species, *Vitis vinifera*, and *Populus trichocarpa* (Yang et al., 2008; Shao et al., 2014; Zhang et al., 2016; Qian et al., 2017). Tandem duplication and dispersed duplication played major roles in the NBS-encoding gene expansion in *X. sorbifolium* and *A. yangbiense* genomes (Table 3). Random dispersed gene duplications and gene losses likely brought out the uneven distributions of these genes on different chromosomes, and this difference was made more apparent through local tandem duplications. This strategy made the NBS-encoding gene form clusters to overcome the limitations of R gene diversity during the coevolution of plants and pathogens (Michelmore and Meyers, 1998; Le Roux et al., 2015).

**FIGURE 6:**
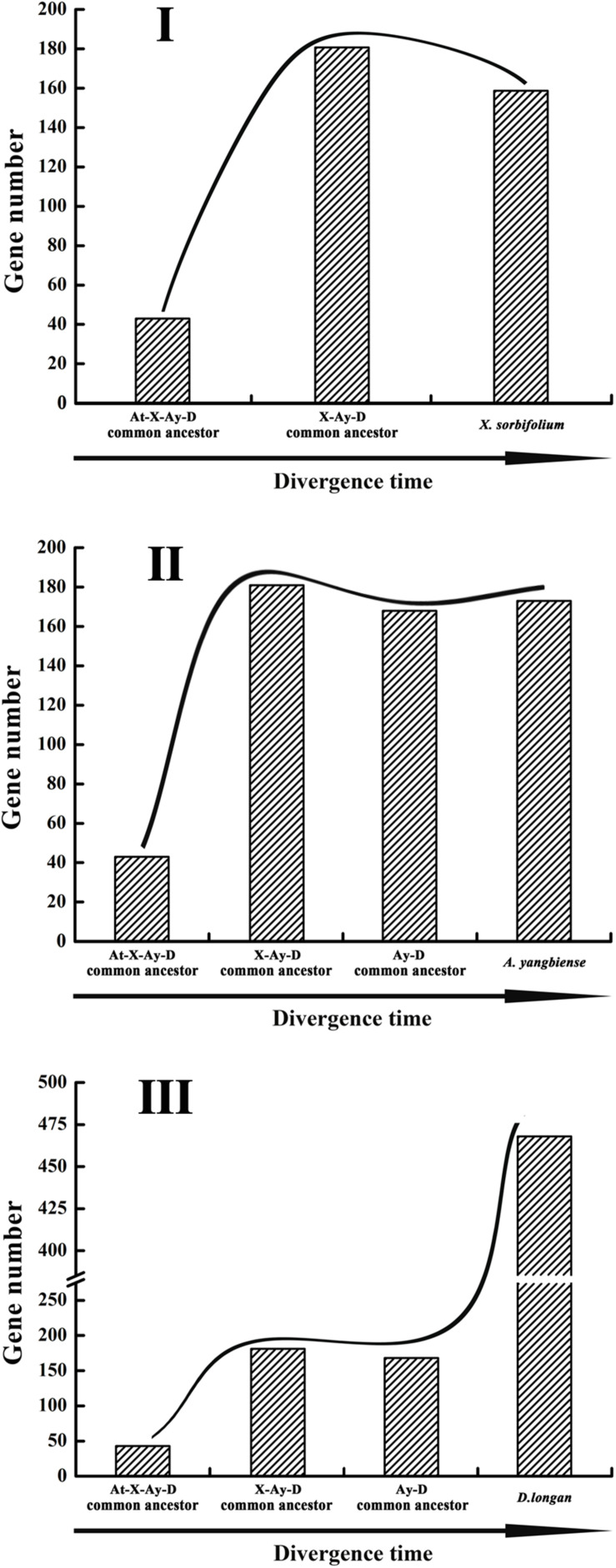
Evolutionary patterns of the NBS-encoding genes in the three Sapindaceae genomes. Gene number variation from the *A. thaliana*, *X. sorbifolium*, *A. yangbiense*, and *D. longan* (At-X-Ay-D) ancestor to *X. sorbifolium* (I), *A. yangbiense* (II), and *D. longan* (III).

The dynamic evolution of NBS-encoding genes in the three Sapindaceae species and *A. thaliana* was traced back to different time periods by reconciling ancient genes in the common ancestor of the three Sapindaceae species and the common ancestor of Sapindaceae and *A. thaliana*. In total, 43 ancient Malvids lineage genes were restored in the common ancestor of Sapindaceae and *A. thaliana* (Figure 2). Among these ancient genes, 10 were lost by the Sapindaceae common ancestor before the three Sapindaceae species diverged. The remaining 33 Malvids lineage genes were inherited and duplicated into 181 genes in the common ancestor of the three Sapindaceae species. These genes experienced further species-specific evolution during speciation and resulted in the currently observed NBS-encoding genes of the three genomes. Over time, the ancient NBS-encoding genes in the common ancestor of Sapindaceae and *A. thaliana* experienced different evolutionary patterns during the speciation of the three Sapindaceae species (Figure 6). *X. sorbifolium* exhibited a “first expansion and then contraction” evolutionary pattern (Figure 6I); more genes were duplicated in the common ancestor of Sapindaceae, but subsequently lost more genes during speciation. Although *A. yangbiense* and *D. longan* exhibited similar evolutionary patterns (Figures 6II,III), expansion followed by contraction and further expansion, subsequent expansion in *D. longan* was stronger and gained more genes than *A. yangbiense* after their divergence (Figure 5A). Furthermore, the evolutionary patterns of these three subclasses of NBS-encoding genes were also diverse among the three species. For example, the CNL and RNL genes exhibited similar patterns as the NBS-encoding genes of corresponding species (Figure 5B), while the patterns of TNL genes were altered in *A. yangbiense* (recent further contraction) and *X. sorbifolium* (recent further expansion) (Figure 5B). Such small changes in TNL genes had little effect on the overall trends of the NBS-encoding genes in *A. yangbiense* and *X. sorbifolium* due to the dominance of CNL genes (see discussion below). Therefore, distinct and dynamic evolutionary patterns of the NBS-encoding genes caused by independent gene duplication/loss events appeared during the speciation of the three Sapindaceae species.

### Numbers of Subclass Genes and the Causes

The number of CNL subclass genes in *X. sorbifolium*, *D. longan*, and *A. yangbiense* was considerably higher than TNL and RNL genes, accounting for 81.11, 89.26, and 89.68% of the NBS-encoding genes in each species, respectively. The greater number of CNL subclass genes is a common phenomenon observed among plant species, except for the Brassicaceae (Zhang et al., 2016). For example, CNL subclass genes are the majority in the genome of *Glycine max* (57.8%), *Phaseolus vulgaris* (66.5%), *V. vinifera* (82.8%), *Capsicum annuum* (94.1%), *Solanum lycopersicum* (87.0%), *Solanum tuberosum* (83.7%), and *Amborella trichopoda* (84.8%) (Yang et al., 2008; Shao et al., 2014, 2016; Jia et al., 2015; Qian et al., 2017). Extremely, monocots have only CNL genes due to the loss of TNL genes near dicot/monocot differentiation (Li et al., 2010; Shao et al., 2016). Previously, it was reported that CNL genes underwent twice gene expansions and expanded earlier than TNL genes during angiosperm evolution (Shao et al., 2016). A total of 155 CNL, 23 TNL, and three RNL ancestral genes were reconciled in the common ancestor of the three Sapindaceae species. Therefore, when the common ancestor of the three Sapindaceae species emerged, its genome possessed more CNL genes than TNL or RNL genes. Although these ancestral genes have experienced frequent gene duplication/loss events during the divergence of the three Sapindaceae species, gene expansion in their common ancestor maintained the large number of CNL genes. Additionally, lots of recent expansions of CNL genes in the three Sapindaceae species may have given rise to the dominance of this subclass’s genes (Figures 2, 5 and Supplementary Figure S6). An extremely small number of RNL genes were previously reported in angiosperms (Shao et al., 2016; Zhang et al., 2016; Qian et al., 2017; Xue et al., 2020). In this study, two, six, and nine RNL genes were identified in *X. sorbifolium*, *A. yangbiense*, and *D. longan*, respectively. It was speculated that CNL and TNL genes were involved in recognizing specific pathogens, while RNL genes helped transduce signals in the downstream pathways of plant immunity (Bonardi et al., 2011; Collier et al., 2011; Tamborski and Krasileva, 2020). Therefore, RNL genes clearly participated in basic defense responses and without the divergent selection of pathogens, these genes did not necessarily expand in large numbers. Moreover, pairwise synteny analysis detected more syntenic CNL (74 genes) subclass genes than TNL (two genes) and RNL (one gene) subclass genes among the three Sapindaceae genomes (Figure 4 and Supplementary Table S2). These differences might be caused by the different evolutionary feature of the three subclass genes during angiosperm evolution: long-term contraction and fast evolution rate of TNL genes, gradual expansion of CNL genes, and conservative evolution of RNL genes (Zhang et al., 2011; Shao et al., 2016).

Notably, not all of the identified NBS-encoding genes in *X. sorbifolium*, *A. yangbiense*, and *D. longan* genomes had intact structures possessing all three domains. There were only 81 (24 TNL, 55 CNL, two RNL), 31 (three TNL, 26 CNL, two RNL), and 247 (24 TNL, 218 CNL, five RNL) NBS-encoding genes with intact structures in *X. sorbifolium*, *A. yangbiense*, and *D. longan* genomes (Table 1), respectively, accounting for 45.0, 12.3, and 43.5% of all identified NBS-encoding genes in these three species, respectively. Similarly, the relative small proportion of NBS-encoding genes with intact structures were also reported in *C. annuum* (23.2%), *S. lycopersicum* (42.7%), *S. tuberosum* (28.2%), *P. trichocarpa* (46.2%), *M. truncatula* (39.1%), *Lotus japonicus* (31.0%), and *Oryza sativa* (30.6%) genomes (Yang et al., 2008; Zhang et al., 2011; Shao et al., 2014; Qian et al., 2017). In plants, the highly conserved NBS domain has been demonstrated to be able to bind and hydrolyze ATP or GTP and act as molecular switches in immune signaling, while the LRR and N-terminal domains (TIR and CC) are typically involved in the recognition of, and the activation of, corresponding partners, respectively (Meyers et al., 1999; Dangl and Jones, 2001; Lukasik and Takken, 2009). Theoretically, in order to trigger immune responses and transfer defense signals, an NBS-encoding gene should possess an intact structure. However, transient overexpression results showed that those NBS-encoding genes without intact structures also function in plant immunity (Nandety et al., 2013; Kato et al., 2014).

## Data Availability Statement

The datasets generated for this study can be found in the *X. sorbifolium*: http://dx.doi.org/10.5524/100606, *D. longan*: http://dx.doi.org/10.5524/100276, and *A. yangbiense*: http://dx.doi.org/10.5524/100610.

## Author Contributions

G-CZ, WL, and Y-LW conceived and directed the project. G-CZ, WL, Y-MZ, and YL obtained and analyzed the data. Y-MZ, ML, MZ, YL, and G-QM conducted the phylogenetic analysis and constructed the discussion. G-CZ wrote the manuscript. All of the authors contributed to the discussion of the results, reviewed the manuscript, and approved of the final article.

## Conflict of Interest

The authors declare that the research was conducted in the absence of any commercial or financial relationships that could be construed as a potential conflict of interest.

## Footnotes

1 www.iplant.cn/foc/

2 http://dx.doi.org/10.5524/100606

3 http://dx.doi.org/10.5524/100276

4 http://dx.doi.org/10.5524/100610

5 http://hmmer.org

6 http://pfam.sanger.ac.uk/

7 https://www.ncbi.nlm.nih.gov/Structure/cdd/wrpsb.cgi

8 http://pfam.janelia.org/

9 http://smart.embl-heidelberg.de/

10 https://github.com/CJ-Chen/TBtools

## Supplementary Material

FIGURE S1 Phylogenetic relationship of *X. sorbifolium*, *A. yangbiense*, and *D. longan.* Time of divergence: million years ago (MYA) (Koenen et al., 2015; Liu et al., 2015; Lin et al., 2017; Bi et al., 2019; Yang et al., 2019).

FIGURE S2 Details of the amino acid frequencies of the whole NBS domain of CNL, TNL, and RNL genes in the three Sapindaceae species (WebLogo).

FIGURE S3 The chromosomal distribution of identified NBS-encoding genes in the *X. sorbifolium* genome.

FIGURE S4 The chromosomal distribution of identified NBS-encoding genes in the *A. yangbiense* genome.

FIGURE S5 The detailed ML tree of the NBS-encoding genes. Sequence names and supporting values are presented on all of the nodes. The tree was reconstructed based on the amino acids of the NBS domain of the NBS-encoding genes in *X. sorbifolium*, *A. yangbiense*, and *D. longan* using NBS-encoding genes in the *A. thaliana* genome were used as a reference.

FIGURE S6 The reconciled NBS-encoding gene tree with real species phylogeny and restored various duplication/loss events. “n4” indicates a loss event that occurred in the common ancestor of *X. sorbifolium*, *A. yangbiense*, and *D. longan*. “n2” indicates a loss event that occurred in the common ancestor of *A. yangbiense* and *D. longan.*

TABLE S1 A list of NBS-encoding genes identified from the three Sapindaceae genomes.

TABLE S2 Detailed information of the synteny NBS-encoding genes in three Sapindaceae genomes.
