# Supplementary figures and images for: Distinct Evolutionary Patterns of NBS-Encoding Genes in Three Soapberry Family (Sapindaceae) Species
Source: Front Genet. 2020 Jul 10;11:737. doi: 10.3389/fgene.2020.00737 (PMC7365912; doi:10.3389/fgene.2020.00737)

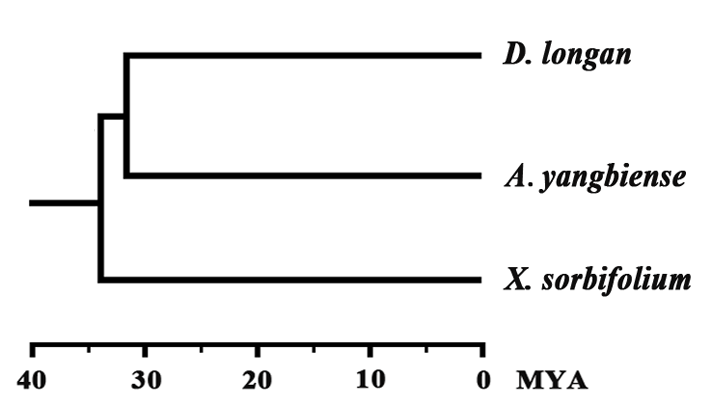

Supplement: FIGURE S1 — Phylogenetic relationship of X. sorbifolium, A. yangbiense, and D. longan. Time of divergence: million years ago (MYA) (Koenen et al., 2015; Liu et al., 2015; Lin et al., 2017; Bi et al., 2019; Yang et al., 2019). [file Image_1.TIF]

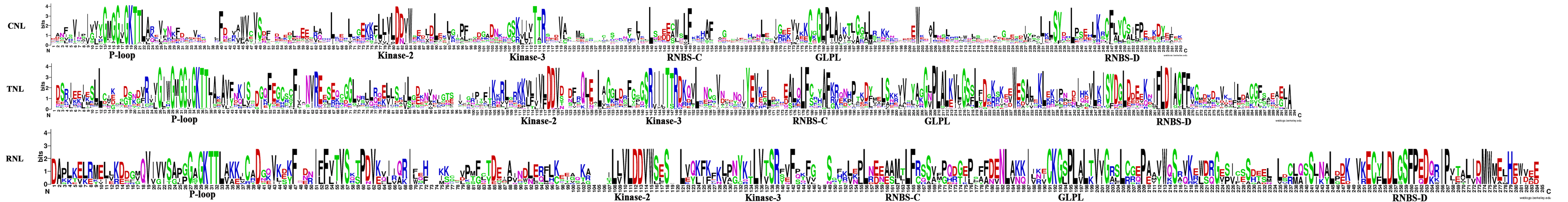

Supplement: FIGURE S2 — Details of the amino acid frequencies of the whole NBS domain of CNL, TNL, and RNL genes in the three Sapindaceae species (WebLogo). [file Image_2.TIF]

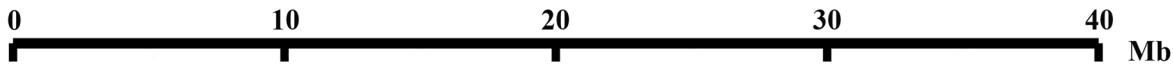

Chrom1

Chrom2

Chrom3

Chrom4

Chrom5

Chrom6

Chrom7

Chrom8

Chrom9

Chrom10

Chrom11

Chrom12

Chrom13

Chrom14

Chrom15

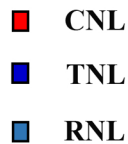

Supplement: FIGURE S3 — The chromosomal distribution of identified NBS-encoding genes in the X. sorbifolium genome. [file Image_3.pdf]

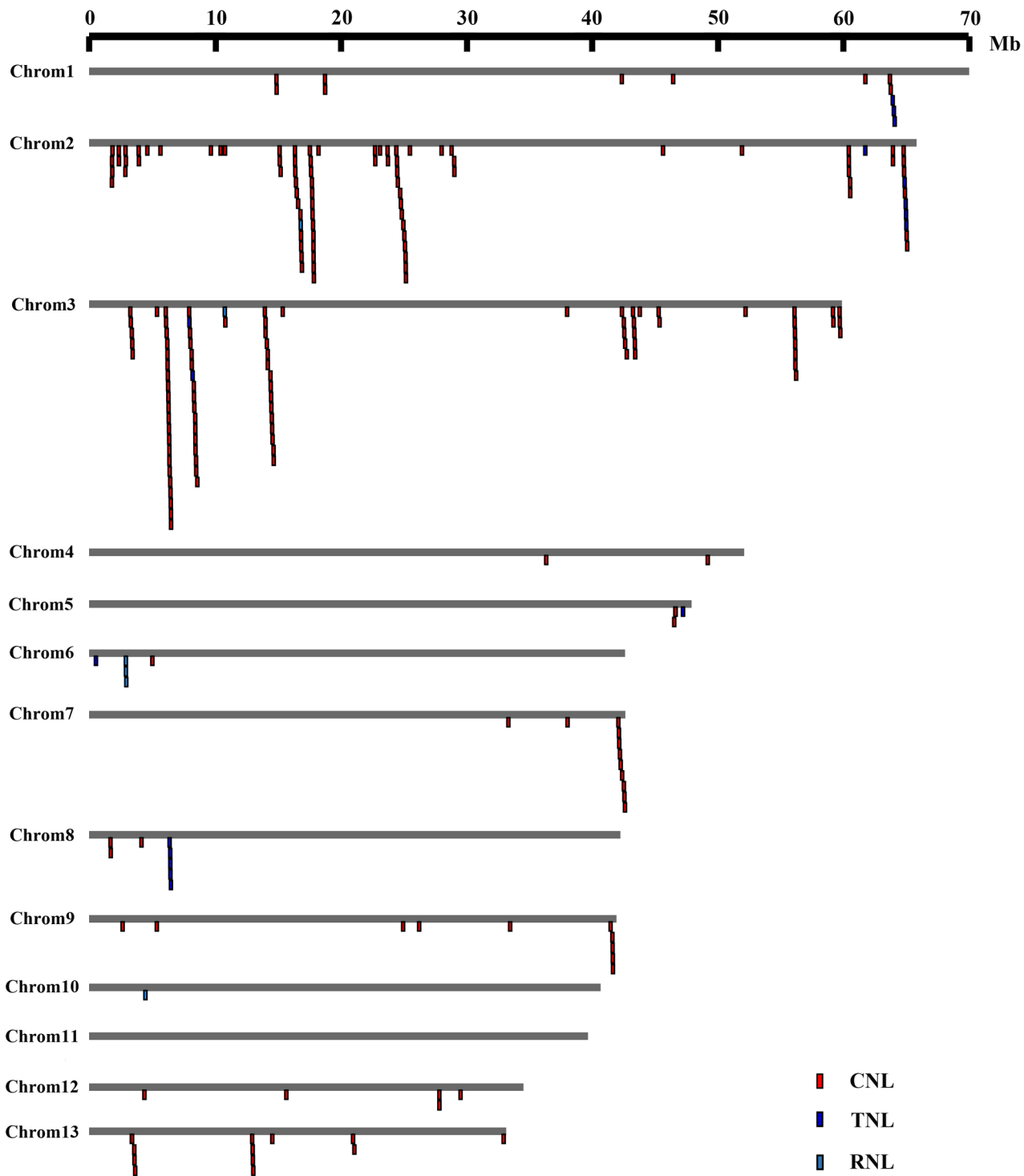

Supplement: FIGURE S4 — The chromosomal distribution of identified NBS-encoding genes in the A. yangbiense genome. [file Image_4.pdf]
