# Supplementary material for: Distinct Evolutionary Patterns of NBS-Encoding Genes in Three Soapberry Family (Sapindaceae) Species
Source: Front Genet. 2020 Jul 10;11:737. doi: 10.3389/fgene.2020.00737 (PMC7365912; doi:10.3389/fgene.2020.00737)
Supplement: FIGURE S5 — The detailed ML tree of the NBS-encoding genes. Sequence names and supporting values are presented on all of the nodes. The tree was reconstructed based on the amino acids of the NBS domain of the NBS-encoding genes in X. sorbifolium, A. yangbiense, and D. longan using NBS-encoding genes in the A. thaliana genome were used as a reference. [file Image_5.pdf]

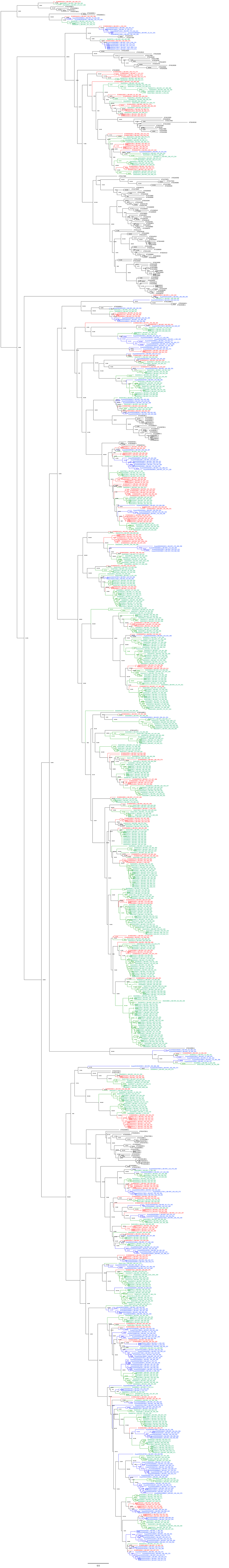
0.5
