# Supplementary material for: Distinct Evolutionary Patterns of NBS-Encoding Genes in Three Soapberry Family (Sapindaceae) Species
Source: Front Genet. 2020 Jul 10;11:737. doi: 10.3389/fgene.2020.00737 (PMC7365912; doi:10.3389/fgene.2020.00737)
Supplement: FIGURE S6 — The reconciled NBS-encoding gene tree with real species phylogeny and restored various duplication/loss events. “n4” indicates a loss event that occurred in the common ancestor of X. sorbifolium, A. yangbiense, and D. longan. “n2” indicates a loss event that occurred in the common ancestor of A. yangbiense and D. longan. [file Image_6.pdf]

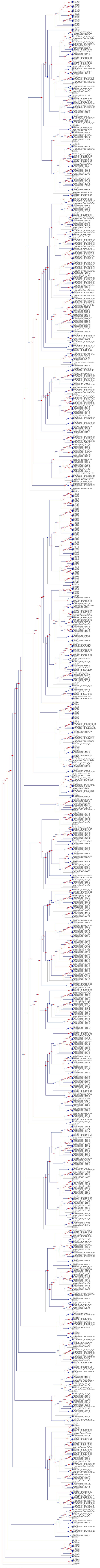

Image generated with Notung 2.6, on 2020-1-19
